# Kinetic Insights into the Antioxidant Effect of Isatin-Thiosemicarbazone in Biodiesel Blends

**DOI:** 10.3390/antiox13070819

**Published:** 2024-07-08

**Authors:** Nalan Türköz Karakullukçu, Halit Muğlu, Hasan Yakan, Volkan Murat Yılmaz, Sarmad Marah, İkbal Agah İnce

**Affiliations:** 1 Karadeniz Advanced Technology Research and Application Center, Ondokuz Mayis University, Atakum, 55200 Samsun, Turkey; 2 Department of Chemistry, Faculty Science, Kastamonu University, 37150 Kastamonu, Turkey; hmuglu@kastamonu.edu.tr; 3 Department of Chemistry Education, Faculty of Education, Ondokuz Mayis University, Atakum, 55200 Samsun, Turkey; hasany@omu.edu.tr; 4 Central Research Laboratory, Bartin University, 74100 Bartin, Turkey; myilmaz@bartin.edu.tr; 5 Department of Chemistry, Faculty of Science, Ondokuz Mayis University, Atakum, 55200 Samsun, Turkey; sarmad159159@gmail.com; 6 Department of Medical Microbiology, School of Medicine, Acibadem Mehmet, Ali Aydinlar University, Atasehir, 34752 İstanbul, Turkey; ikbal.agah.ince@gmail.com

**Keywords:** thiosemicarbazones, biodiesel, oxidative stability, kinetic study, energy

## Abstract

Biodiesel has several drawbacks, such as being prone to oxidation, having reduced stability, and having limited storage time. Antioxidants compatible with biodiesel are being used to address its drawbacks. Utilizing antioxidants effectively improves the quality of biodiesel. Enhancing the quality of biodiesel for use as a clean energy source benefits both the global economy and ecology. Therefore, we believe that our work will contribute to the advancement of the biodiesel industry worldwide. This study used blends consisting of 20% biodiesel and 80% diesel fuel. Isatin-thiosemicarbazones were tested as additives in blends at a concentration of 3000 parts per million (ppm) using an oxifast device and were compared with the chemical antioxidant Trolox. FT-IR, DSC, and TGA were used to characterize these samples. DSC measured sample crystallization temperatures (Tc). Samples with antioxidants showed decreased values compared to the non-antioxidant diesel sample D100. Several DSC tests were conducted to determine the antioxidant strengths of various samples. The results show that the FT-IR spectrum’s antioxidant effect regions grow clearer with antioxidants. The extra antioxidant is effective. Biodiesel’s oxidative stability improves with isatin-thiosemicarbazones at varying concentrations. The kinetics of thermal decomposition of isatin-thiosemicarbazones under non-isothermal conditions were determined using the Kissinger, Ozawa, and Boswell techniques. The activation energies of compounds **1** and **2** were calculated as 137–147 kJ mol^−1^ and 173–183 kJ mol^−1^, respectively.

## 1. Introduction

Antioxidants are a group of molecules that effectively halt the process of oxidation. In order to effectively manage the oxidation of biodiesel, one method employed is the inhibition of free radical formation or the scavenging of existing radicals [1]. Antioxidants are commonly composed of phenolic functional groups within their chemical structure [2]. In recent studies, it has been discovered that the addition of both natural and synthetic antioxidants to biodiesel is crucial for improving and maximizing its oxidative stability. The significance of reducing the number of free radicals in biodiesel and delaying oxidation is highlighted by the use of antioxidants [3]. The auto-oxidation mechanism involves the interruption of the progression of the peroxide radical (ROO˙.) by the antioxidant (AH), which inhibits the creation of additional radicals. The oxidation stability of biodiesel can be improved by incorporating antioxidants in specific proportions. This addition serves to prevent the occurrence of oxidative reactions with antioxidant radicals [4]. The addition of antioxidants to biodiesel has been found to significantly improve its overall quality, extend its storage time, and enhance its durability [5].

Predictions indicate that there will be an increase in the utilization of fossil fuels to meet the growing energy demand. In recent years, there has been a growing body of research dedicated to exploring alternative fuel sources as a response to the detrimental effects of fossil resource consumption on environmental contamination [6]. The utilization of biodiesel offers a wide range of advantages. The non-toxic nature of this specific characteristic provides both safety and cost-effectiveness, making it a significant advantage [7]. The production of biodiesel is a complex process that involves several chemical reactions. These reactions lead to the formation of free radicals, which are highly reactive molecules that can easily undergo oxidation when exposed to the surrounding atmosphere [8]. In one study, it was shown that the oxidation process has a negative impact on both fuel efficiency and engine performance [9]. The storage stability of biodiesel is crucial in assessing its overall quality. The significance of standards and fuel quality cannot be overstated [10]. The study of heterocyclic Schiff bases and their metallic complexes is of significant interest in the fields of chemistry, biology, and pharmacology [11,12]. These compounds are formed through the primary amino groups of ketones or the condensation reaction of aldehydes [11,13]. They exhibit various biological functions, including antioxidant [14], antifungal [14], antitumor [15], anticancer [16], antiviral [17], antimicrobial [18], and antimalarial properties [19]. Phenolic Schiff bases (SB-OH) exhibit antioxidant activity that is directly linked to their capacity to release hydrogen atoms [20]. Schiff bases have the potential to enhance the quality of biodiesel–diesel blends through two distinct mechanisms. Firstly, they can form complexes with metal ions, effectively deactivating them. Secondly, they can trap radicals present in biodiesel–diesel blends, thereby interrupting the chain reaction of radical reactions. Schiff bases are prone to undergoing reactions due to the presence of a nitrogen atom in the azomethine group. The nitrogen atom is in the sp^2^ hybrid orbital and possesses a lone electron pair [21]. Schiff bases have gained considerable prominence in the academic world due to their exceptional chelating powers. Trace amounts of some metals in diesel fuel have been seen to act as catalysts, accelerating the reactions related to fuel instability. Metal deactivators work by forming chelates with metals, which efficiently deactivate their catalytic activity [22,23].

Furthermore, the presence of Schiff bases in biodiesel can efficiently regulate the oxidation process by either preventing the generation of free radicals or scavenging existing radicals, even in low concentrations. Antioxidants often contain phenolic functional groups in their chemical composition. They provide a notable decrease in the breakdown of biodiesel. The use of antioxidants highlights the significance of reducing the presence of free radicals in biodiesel and extending the oxidation process. Phenolic antioxidants (AH) are commonly used as scavengers of free radicals because of their beneficial characteristics [24,25].

Phenolic compounds commonly exhibit the ability to scavenge free radicals. The antioxidative activity of phenolic Schiff bases (SB-OH) is strongly linked to their ability to release hydrogen atoms. Several techniques can be employed to eliminate free radicals, such as hydrogen atom transfer (HAT), single electron transfer followed by proton transfer (SET-PT), and sequential proton loss electron transfer (SPLET). Each of these procedures yields the identical result, namely the production of the suitable phenoxy radical. The hydrogen atom transfer (HAT) process is defined by a solitary step in which a hydrogen atom undergoes conversion into a free radical, as given in Reaction (1).

(1) $$\mathrm{SB}-\mathrm{OH}\to {\mathrm{SBO}}^{•}+H^{•}$$

The SET-PT and SPLET mechanisms consist of a two-step procedure. The SET-PT process starts with electron loss, leading to the formation of a radical cation. Afterwards, in the second stage, the radical cation experiences deprotonation, resulting in the creation of a matching radical, as given in Reaction (2).

(2) $$\begin{array}{l} \mathrm{SB}-\mathrm{OH}\to {\mathrm{SB}-\mathrm{OH}}^{•+}{+e}^{-}\\ {\mathrm{SB}-\mathrm{OH}}^{•+}\to {\mathrm{SB}-O}^{•}{+H}^{+} \end{array}$$

The first stage in the SPLET process involves the removal of a proton from the parent molecule. In the second step, the anion experiences electron loss, leading to the creation of a corresponding radical, as given in Reaction (3) [20,21,22].

(3) $$\begin{array}{l} \mathrm{SB}-\mathrm{OH}\to {\mathrm{SB}-O}^{-}{+H}^{+}\\ {\mathrm{SB}-O}^{-}\to {\mathrm{SB}-O}^{•}{+e}^{-} \end{array}$$

Thiosemicarbazones are a notable category of synthetic chemicals that exhibit a diverse range of pharmacological and biological activities [26]. A diverse range of actions have been disclosed, including anti-HIV [27], anticancer [28], antimalarial [29], anticonvulsant [30], anti-inflammatory [31], antioxidant [32], anti-viral [33], enzymatic inhibition [34], antifungal [35], and antibacterial [36] properties. Isatin derivatives have demonstrated a wide range of biological activities, including antioxidant [37], antiviral [38], antifungal [39], antibacterial [40], antimicrobial [41], anti-HIV [27], antitubercular [42], and anticonvulsant [43] properties. In this study, two previously synthesized isatin-thiosemicarbazones were used, whose chemical structures were elucidated by means of spectroscopic methods. Schiff bases have potent antioxidant and metal-chelating effects. Schiff base-based isatin-thiosemicarbazone derivatives have been preferred as substitutes for organic and synthetic antioxidants due to their ability to counteract radical reactions in biodiesel–diesel fuel mixes [21]. Using a comparative analysis with Trolox, the antioxidant capacity of the compounds employed was confirmed [44]. Trolox facilitates high-throughput screening for putative antioxidant capacity [45]. These approaches are employed to evaluate the antioxidant capacity of biological samples such as plasma, individual chemicals, dietary components, or food extracts [40,41]. However, it is believed that this synthetic antioxidant variant possesses hazardous and carcinogenic properties. Isatin-thiosemicarbazone derivatives were added, and a concentration of 3000 parts per million (ppm) was added to the mixtures. Several characterization techniques, including thermogravimetric analysis (TGA), differential scanning calorimetry (DSC), Fourier-transform infrared spectroscopy (FT-IR), and the 1,1-diphenyl-2-picryl hydrazyl (DPPH˙) assay were used to evaluate the antioxidant activity of isatin-thiosemicarbazone derivatives. Also, the investigation of the breakdown kinetics of isatin-thiosemicarbazone derivatives was conducted using TGA. Non-isothermal experiments are more appropriate than isothermal kinetic studies for avoiding a rapid increase in temperature in the sample at the start. Conventional techniques that include fitting experimental data to the reaction model are not effective in providing accurate kinetic information for non-isothermal research. This is because these approaches fail to properly differentiate between the reaction model, f(α), and the temperature dependency, k(T). To address this drawback of model fitting, one might employ model-free isoconversional approaches. These approaches enable the determination of the activation energy based on conversion or temperature without making any assumptions about the reaction model [46].

## 2. Materials and Methods

### 2.1. Materials

Compounds from recognized suppliers such as Merck, Sigma, (St. Louis, MO, USA) or Aldrich Chemical Company (St. Louis, MO, USA) were used without further purification in their as-received form. In the methods used to characterize the obtained Schiff bases, the solvent used was of spectroscopic quality. An elemental analysis was conducted using a CHNS-932 instrument (LECO, St. Joseph, MI, USA). The Bruker Alpha FT-IR spectrometer was used to record infrared spectra. The JEOL ECX-400 (400 MHz) spectrophotometer was used to obtain ^1^H NMR and ^13^C NMR spectra in DMSO-*d*_6_. The melting points were determined with Gallenkamp melting point equipment. Thermal investigations of isatin-thiosemicarbazone compounds were carried out utilizing a Hitachi STA 7300 Thermogravimetric Analyzer (TGA).

The methods used to measure the ability of the resulting mixtures to exhibit antioxidant activity were as follows:

The thermogravimetric analysis (TGA) was performed using SDTQ 600, while the differential scanning calorimetry (DSC) was conducted using DSCQ 2000. Fourier-transform infrared spectroscopy (FT-IR) was carried out using ATR-FTIR (Perkin Elmer, Spectrum-Two, Waltham, MA, USA).

Diesel fuel (D100) was given to OPET in Samsun, Turkiye, while biodiesel (B100), made entirely from waste sunflower and corn oil, was given to Aves Energy Oil and Food Industry in Mersin, Turkiye.

### 2.2. Methods

#### 2.2.1. Schiff Based Isatin-Thiosemicarbazones Synthesis

**Figure 1:**
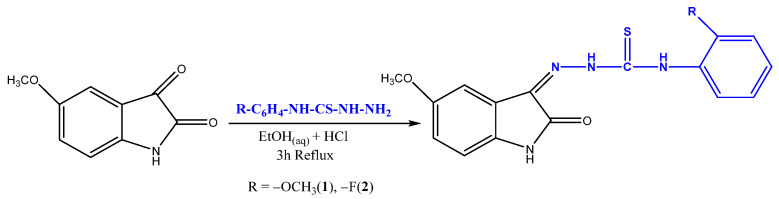
Synthetic route for Schiff bases based on isatin-thiosemicarbazones (**1**–**2**).

The configuration of the substituents in the thiocarbohydrazone is vital in defining its antioxidant activity, due to the role of the substituents in the carbohydrazone structure [47]. A previous work carried out by Kiran et al. [48] found that the presence of halogen-bonded aromatic structures in novel bis-isatin carbohydrazone derivatives was responsible for the antioxidant activity. The isatin-thiosemicarbazones chosen for this study were produced by reacting 5-methoxyisatin with thiosemicarbazides in a mixture of aqueous ethanol and one drop of hydrochloric acid. The reaction was carried out at reflux for a duration of three hours (Figure 1). The synthesis and characterization of the compounds have been performed previously by Muğlu [49]. IR, ^1^H NMR, and ^13^C NMR data as well as elemental analysis were utilized to confirm the chemical structures of products. The physical data, melting points, and yields of the compounds are summarized in Table 1.

*N*^4^-2-methoxyphenyl-5-methoxyisatin-*β*-thiosemicarbazone (**1**) Yield: 87%, m.p.: 218–220 °C, IR (KBr, cm^−1^): ν(–NH ist) 3207, ν(–C=N–NH and –NH–Ar carbazide) 3137, 3092, ν(Ar C–H) 3043–2995, ν(C=O) 1687, ν(C=N) 1544, ν(C=S) 1487, ν(C–N) 1182; ^1^H NMR (400 MHz, DMSO-d_6_) δ/ppm: 12.71 (s, NH ist), 11.01 (s, NH), 10.39 (s, NH), 7.67–7.65 (d, 1H, *J* = 7.9 Hz), 7.28–7.23 (m, 2H, ArH), 7.10–7.08 (d, 1H, *J* = 7.9 Hz), 6.98–6.94 (t, 1H, *J* = 7.6 Hz), 6.92–6.89 (dd, 1H, *J* = 8.5, 2.4 Hz, ArH), 6.83–6.81 (d, 1H, J = 8.5 Hz, ArH), 3.79 (s, 3H, OCH_3_), 3.71 (s, 3H, OCH_3_); **^13^**C NMR (100 MHz, DMSO-d_6_) δ/ppm: 177.07 (C=S), 163.24 (C=O), 155.84 (C–O), 153.90 (C–O), 136.79 (C=N), 132.97, 128.35, 127.55, 127.51, 121.13, 120.65, 118.15, 112.47, 112.34, 106.65, 56.28 (OCH_3_), 56.08 (OCH_3_); Elemental Analysis Calcd: C, 57.29; H, 4.53; N, 15.72. Found: C, 56.84; H, 4.61; N, 15.34.

*N*^4^-2-fluorophenyl-5-methoxyisatin-*β*-thiosemicarbazone (**2**) Yield: 82%, m.p.: 234–235 °C, IR (KBr, cm^−1^): ν(–NH ist) 3309, ν(–C=N–NH and –NH–Ar carbazide) 3147, 3096, ν(Ar C–H) 3040–3005, ν(C=O) 1686, ν(C=N) 1536, ν(C=S), 1479, ν(C–N) 1193, ν(C–F) 906; ^1^H NMR (400 MHz, DMSO-d_6_) δ/ppm: 12.81 (s, NH ist), 11.02 (s, NH), 10.64 (s, NH), 7.47–7.43 (t, 1H, *J* = 7.3 Hz), 7.39–7.34 (m, 1H, ArH), 7.32–7.30 (d, 2H, *J* = 7.9 Hz), 7.27–7.21 (m, 1H, *J* = 7.7 Hz, ArH), 6.92–6.89 (dd, 1H, *J* = 8.5, 2.4 Hz, ArH), 6.83–6.81 (d, 1H, *J* = 8.5 Hz, ArH), 3.71 (s, 3H, OCH_3_); **^13^**C NMR (100 MHz, DMSO-d_6_) δ/ppm: 178.54, 163.29, 159.11, 156.64, 155.86, 136.82, 133.51, 130.80, 129.74, 129.66, 126.98, 126.86, 124.99, 124.96, 121.27, 121.16, 118.23, 116.65, 116.45, 112.47, 106.91, 56.11; Elemental Analysis Calcd: C, 55.81; H, 3.81; N, 16.27. Found: C, 55.44; H, 3.79; N, 16.05.

#### 2.2.2. Preparations of Biodiesel–Diesel Blends

In diesel and biodiesel blends, the ratio of biodiesel to diesel typically ranges from 20% to 80%. The formula B20D80 represents the blended mixture [43]. A recent study carefully blended the additives, Trolox, **2**, and **1**, involved in biodiesel, at a concentration of 3000 ppm. This concentration was chosen to ensure optimal effectiveness and maximize the potential benefits of the combined extracts. The aim was to investigate the potential synergistic effects that could arise from this combination, as each plant extract may possess unique bioactive compounds [44]. To fully understand the implications and potential applications of Table 2, further research is necessary. Various sample codes and content were presented.

The ratio of biodiesel to diesel in diesel and biodiesel blends typically ranges from 20% to 80%. The mixture being blended is represented by the formula B20D80 [50]. A recent study carefully blended the additives, Trolox, **2**, and **1**, involved in biodiesel, at a concentration of 3000 ppm. This concentration was chosen to ensure optimal effectiveness and maximize the potential benefits of the combined additives [51]. Further research is needed to fully understand the implications and potential applications of Table 2. Various sample codes and content were presented.

#### 2.2.3. Differential Scanning Calorimetry (DSC)

DSC approaches can be used to characterize, quantify, and infer the precise moment when crystallization begins in fuel samples. After taking all these factors into account, it can be concluded that the chemical exhibits traits like quick oxidation, early crystallization, and heightened susceptibility to oxidation. This decline results in a decrease in the stability of the characteristics. The primary objective of this study was to investigate the temperatures at which crystallization begins (measured in degrees Celsius) for the materials D100, B30D70, B30D70BHT, B30D70–**2**, and B30D70–**1**. The experiment was carried out with a TA Q-2000 calorimeter equipped with an RCS90 and a cooling system. Aluminum pans were utilized for the purpose of conducting analysis. In the course of this experimental process, a sample with a weight of 5 ± 0.5 mg was carefully positioned within the pan. The cooling rate throughout the temperature range of 25 °C to −90 °C was determined to be 10 °C per minute, while a nitrogen flow rate of 50 mL per minute was maintained [52].

#### 2.2.4. Thermogravimetric Analysis (TGA)

TGA is commonly employed to investigate the correlation between changes in mass and temperature variations, either rising or constant, within controlled air circumstances. This analytical methodology is utilized to quantify vapors, analyze combustion reactions, examine degradation processes, and ascertain residual components in products. The TGA technique has been employed to observe the decomposition characteristics of various organic compounds and the potential improvement of their constituents. TGA was performed using a SDT Q-600 instrument TA Instruments, New Castle, DE, USA). The collection of samples was achieved by subjecting five powder samples, each weighing 5 ± 0.5 mg, to a heating process at a rate of 10 °C per minute. This heating process took place in an alumina pan, and an oxygen gas flow of 50 mL per minute was maintained during the process. The temperature was increased until it reached 400 °C, as documented in [53]. The DSC and TGA graphics were generated using the Advantage/Universal Analysis (UA) Software, Version 4.5A Build 4.5.0.5 (TA Instruments, New Castle, DE, USA).

Thermal studies of isatin-thiosemicarbazone compounds were conducted using a Hitachi STA 7300. TGA, DTG, and DTA curves were obtained by measuring the thermal behavior of 3 mg samples at a heating rate of 10 °K min^−1^. The experiment was conducted within a temperature range of 25–900 °C in a nitrogen environment with a flow rate of 100 mL min^−1^. A crucible made from Al_2_O_3_ was utilized for conducting measurements [54].

#### 2.2.5. Fourier Transform Infrared Spectroscopy (FT-IR)

FT-IR (Perkin Elmer, Spectrum-Two, USA) was used to analyze the chemical functional groups in isatin-thiosemicarbazone derivatives. The spectral region ranging from 650 to 4000 cm^−1^ was utilized for the purpose of scanning the surface of the sample. The ATR FT-IR spectra were obtained using an isothermal method at room temperature. At the beginning of our work, the background removal techniques, baseline correction, and data tune-up correction were implemented according to the procedure in [55].

#### 2.2.6. DPPH^.^ Method for Antioxidant Activity

The DPPH˙ radical scavenging activity test was used to find out how effective the produced chemicals were as antioxidants. The free radical scavenging ability was assessed using the method of Brand-Williams et al. [56], with minor modifications. To assess antioxidant activity, the compounds were prepared in DMSO at a concentration of 250 µM. The compounds were dissolved in DMSO and then diluted to five different concentrations. The concentration of DPPH˙ in ethanol was 50 µM. Different concentrations of compound solutions (5 µM, 10 µM, 20 µM, 50 µM, and 100 µM) were added to a previously prepared 5 mL DPPH˙ solution, along with enough ethanol to make a total volume of 6 mL. The mixture was measured at 517 nm against a blank after being left for 30 min at room temperature in a dark room [57].

#### 2.2.7. Kinetics of Isatin-Thiosemicarbazone Derivatives

The values of the kinetic parameters reveal certain relationships that contribute to enhanced antioxidant properties. This is attributed to the low activation energy and the appropriate conversion dependence [58]. The degradation of solids may be analyzed using a non-isothermal kinetic approach known as the model-free method. This method involves examining the temperature values associated with the reaction rate (*α*) in thermal analysis graphs obtained at various heating rates. Activation energies can also be calculated using this approach [59]. The thermal decomposition of isatin-thiosemicarbazone derivatives is a process that occurs in the solid state. The kinetics of this reaction may be broadly characterized by Equation (4):

(4) $$\frac{d\left(a\right)}{d(t)}=k\left(T\right)\left(\alpha \right)\;\;\;\;\;\;\;\;\;\;\;\;\;\;\;\;\;\;\;$$

The variables in this equation are as follows: *t* represents the reaction time in minutes, T represents the temperature in Kelvin, *α* represents the degree of conversion or reaction rate, *k*(*T*) represents the rate constant that depends on temperature, and *f*(*α*) represents the unique reaction model. The expression for *k*(*T*) can be represented using the Arrhenius Equation (5):

(5) $$k\left(T\right)=A.\exp\left(\frac{-E}{RT}\right)\;\;\;\;\;\;$$

where *A* represents the exponential factor with units of min^−1^, *R* represents the gas constant with units of 8.314 J mol^−1^ °K^−1^, and *E* represents the activation energy with units of J mol^−1^. The kinetic triples, consisting of the *E*, *A*, and *f*(*α*) values, are used to forecast the conversion degree of a chemical given the temperature [60].

(6) $$\ln\;\left(\frac{\beta }{T^{2}}\right)=\ln\;\left[\frac{E.A}{R.g(a)}\right]-\left(\frac{E}{RT}\right)$$

(7) $$g\left(a\right)=\int_{0}^{a}{f(a)}^{-1}\;da\;\;\;\;\;\;\;\;$$

The activation energies for various *α* values may be determined by calculating the slope of the graph ln (*β*/*T*^2^) versus (1000/*T*) [61], using Equations (6) and (7).

(8) ln ⁡βT2=k1−ERT 

(9) ln ⁡βT=k2−ERT 

(10) ln ⁡β=k3−1051 ERT 

where *k*_1_, *k*_2_, and *k*_3_ are fixed values. The activation energy was determined from the thermogravimetric curves using the Kissinger (Equation (8)), Boswell (Equation (9)), and Ozawa (Equation (10)) techniques [46]. The thermogravimetric analysis tests were conducted using a Hitachi STA 7300 (Hitachi High-Tech Corporation, Tokyo, Japan) at various heating rates (*β* = 5, 10, 15, 20 °K min^−1^) in a N_2_ environment with a flow rate of 100 mL min^−1^. The temperature range for the studies was from ambient temperature to 1173 °K.

#### 2.2.8. Analytical Statistics

The SPSS 22.0 (Statistical Packages for the Social Sciences) package application was used to present the DPPH˙ test findings. The Tukey HSDa, b test was used to produce a one-way ANOVA to compare the differences between the compounds. The findings were expressed as mean ± standard deviation, and *p* < 0.05 indicated statistical significance.

## 3. Results and Discussion

### 3.1. Differential Scanning Calorimetry (DSC)

**Figure 2:**
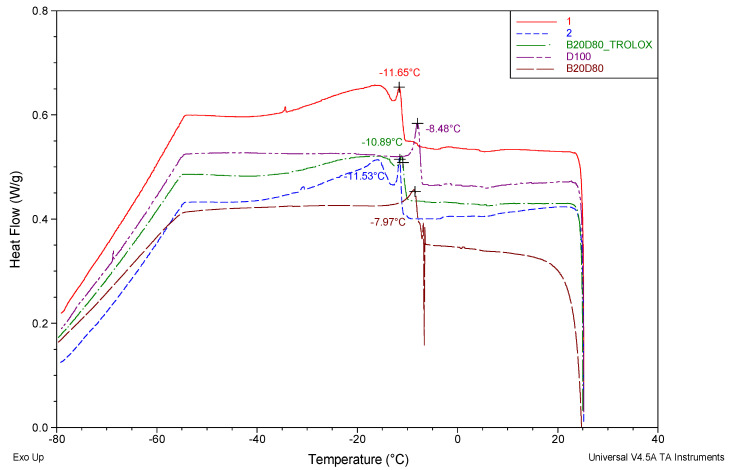
DSC thermograms of D100, B20D80, B20D80TROLOX, B20D80-**2**, and B20D80-**1** under N_2_ atmosphere.

DSC measurements were conducted on a blend of fuel containing isatin-thiosemicarbazone derivatives and unbleached fuel in a nitrogen gas atmosphere. The resulting data are graphically shown in Figure 2.

The purpose of this research was to examine the effects of adding isatin-thiosemicarbazone derivatives to biodiesel–diesel blends on their crystallization temperatures. As a result, this study demonstrated that the extract derived from isatin-thiosemicarbazone derivatives had an increase in the crystallization point. This inquiry pertains to the initiation temperatures of crystallization for four distinct alloys, namely D100, B20D80, B20D80TROLOX, B20D80–**2**, and B20D80–**1**. After conducting the experiments, the starting temperatures for crystallization were found to be −7.97 °C, −8.48 °C, −10.89 °C, −11.53 °C, and −11.65 °C for the samples marked as D100, B20D80, B20D80TROLOX, B20D80–**2**, and B20D80–**1**, respectively. The mean onset temperature for crystallization is $x_{mean}$ = 10.10. A value of 1.75 was determined for the standard deviation, which is a common way to measure the variability of experimental measurements according to Equation (11), ass shown in Table 3.

(11) $$\;\;\;\;\;\;s^{2}=\frac{{\sum_{i=1}^{N}\left(xi-x_{mean}\right)}^{2}}{N-1}\;$$

### 3.2. Thermogravimetric Analysis (TGA)

**Figure 3:**
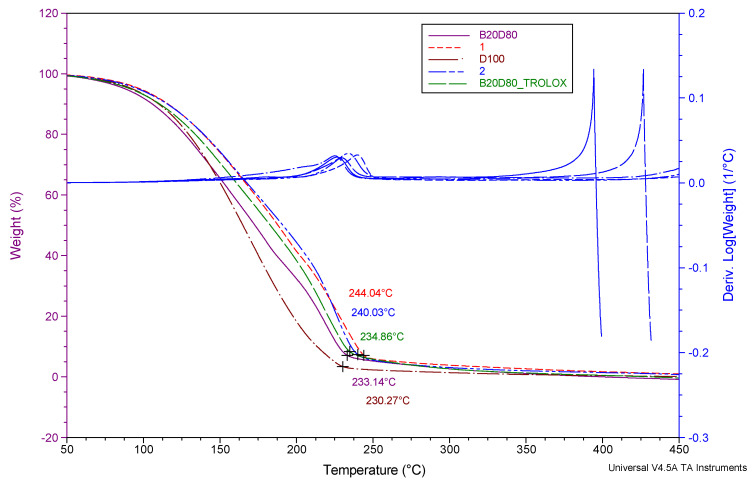
TGA and DrTGA curves of D100, B20D80, B20D80TROLOX, B20D80-**2**, and B20D80-**1** under O_2_ atmosphere.

TGA is a method used to analyze the thermal and oxidative stability of a substance or combination by observing alterations in its physicochemical properties. The properties were described by how the weight changed as the temperature increased. Oxidative stability is an important quality indicator for esters. Thermogravimetric curves (TGA) display the changes in weight as temperature varies, along with the corresponding derivative curves that indicate the rate of weight loss [62]. TGA–DTG compound graphs of several combinations were compared. It is observed that there is only one degree of degradation concerning temperature. The temperature at which degradation begins, known as the onset temperature, offers valuable information regarding thermal stability and the first boiling point. A positive association has been seen between the stability of the samples and the Tonset values, suggesting that an increase in sample stability is associated with a corresponding rise in Tonset values [53]. Figure 3 displays the graphical representations of the thermogravimetric analysis (TGA) and derivative thermogravimetric analysis (DrTGA) curves. Upon closer inspection, the curves are relatively similar to one another. At these temperatures, sample mass is lost at a rate between 98.56 and 99.36%. The values taken from the thermometers and recorded on the thermograms are listed in Table 4.

The mean degradation temperature (Tonset) is $y_{mean}$ = 99.11, with a standard deviation of s = 0.32. The mean mass loss is $z_{mean}$ = 9.22, with a standard deviation of s = 1.65, as calculated using Equation (8), and as shown in Table 4.

### 3.3. Fourier Transform Infrared Spectroscopy (FT-IR)

**Figure 4:**
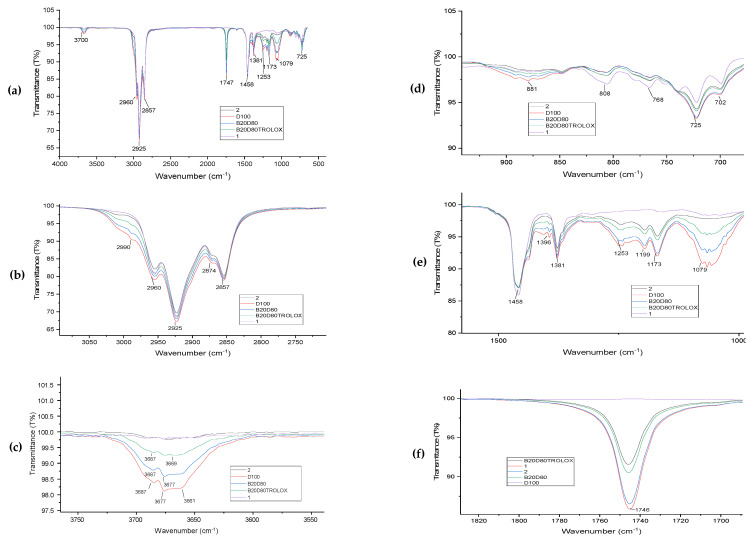
(**a**) FT–IR spectra of D100, B20D80, B20D80TROLOX, B20D80–**2**, and B20D80–**1** at 4000–500 cm**^−^**^1^. (**b**) Antioxidant effects of D100, B20D80, B20D80TROLOX, B20D80–**2**, and B20D80–**1** samples between wavenumbers 2750 and 3050 cm**^−^**^1^. (**c**) Antioxidant effects of D100, B20D80, B20D80TROLOX, B20D80–**2**, and B20D80–**1** samples between wavenumbers 3550 and 3750 cm**^−^**^1^. (**d**) Antioxidant effects of D100, B20D80, B20D80TROLOX, B20D80–**2**, and B20D80–**1** samples between wavenumbers 700 and 900 cm**^−^**^1^. (**e**) Antioxidant effects of D100, B20D80, B20D80TROLOX, B20D80–**2**, and B20D80–**1** samples between wavenumbers 1000 and 1500 cm**^−^**^1^. (**f**) Antioxidant effects of D100, B20D80, B20D80TROLOX, B20D80–**2**, and B20D80–**1** samples between wavenumbers 1700 and 1820 cm**^−^**^1^.

The FT-IR spectra of the biodiesel–diesel blends are depicted in Figure 4a–f. The impact of incorporating antioxidants at a concentration of 3000 ppm into biodiesel samples is displayed in Table 5. The valence-stretching vibration frequency range of an unbounded hydroxyl group, ν(O–H), is approximately 3661–3687 cm^−1^. Spectral peaks in the range of 2925–3000 cm^−1^ are often associated with the vibration of ν(C–H) bonds, indicating oxidation. Aldehyde and ketone compounds in biodiesel samples contain these bonds, which are also found in antioxidants. The spectral range of 2925–2990 cm^−1^ is often linked to the ν(C–H) bending vibration, which is known to be involved in oxidation processes. Peaks in the 1079 cm^−1^ wavenumber indicate stretching of the ν(C–O) bond. The spectrum shows strong absorption bands, indicating the presence of the ester carbonyl functional group (C=O). The absorption is observed in the spectral range of 1746 cm^−1^. The lack of a nearby band could suggest that carboxylic acids are not present. The bending vibrations of ν(N–H) are attributed to the observed vibrational modes at 1458 cm^−1^. In isatin-thiocarbohydrazones, the bending vibrations of ν(C–N) occur within the range of 1199–1173 cm^−1^. The long-term stability of biodiesel samples is linked to low oxidation levels [54]. Antioxidants, particularly phenolic chemicals, have been found to enhance peak intensity.

### 3.4. DPPH˙ Free Radical Scavenger Effect for Isatin-Thiosemicarbazones

The study assessed the samples’ capacity to eliminate free radicals. Compound **1** had more activity, with a value of 66.178 ± 0.11 μM, whereas compound **2** showed lesser activity, with a value of 79.927 ± 0.13 μM. The arrangement of substituents in isatin-thiocarbohydrazones plays a vital role in determining their antioxidant activity, which is comparable to that of carbohydrazones [63]. The antioxidant activity of isatin-thiosemicarbazone derivatives can be attributed to the presence of substituted groups/atoms attached to the aromatic ring in Table 6.

### 3.5. Thermal Characterization of Isatin-Thiosemicarbazones

**Figure 5:**
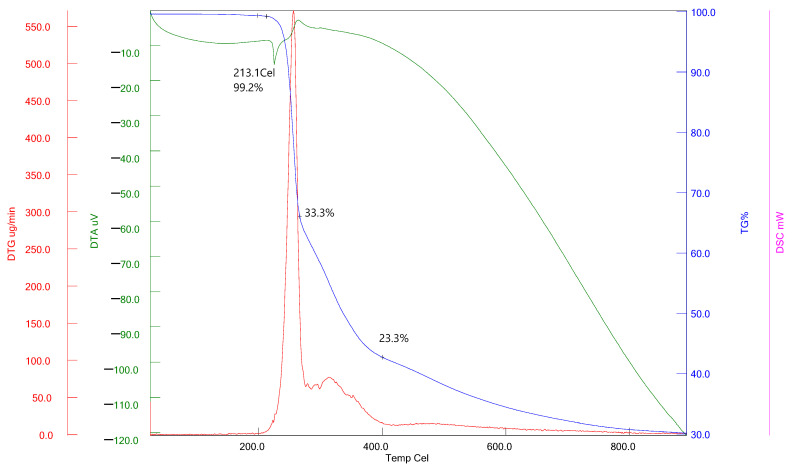
Studying the thermal properties of compound **1.**

Compound **1** is represented by the TGA, DTG, and DTA curves in Figure 5. The thermogravimetric (TGA) curve revealed that the thermal decomposition of compound **1** commenced at a temperature of 201 °C and exhibited two distinct stages of weight loss. The first and subsequent stages of weight loss account for 33.3% and 23.3%, respectively. The weight loss experienced from room temperature to 900 °C is precisely 69.8%. The differential thermogravimetric (DTG) curve of compound **1** exhibited two distinct peaks that corresponded to the thermogravimetric (TGA) curve. The initial stage was characterized by an exothermic peak at 213.1 °C observed in the DTA graph, indicating the melting point of the sample. The second stage was detected at a temperature of 265 °C on the DTA graph, and it manifested as an exothermic peak, indicating the thermal decomposition of compound **1**.

**Figure 6:**
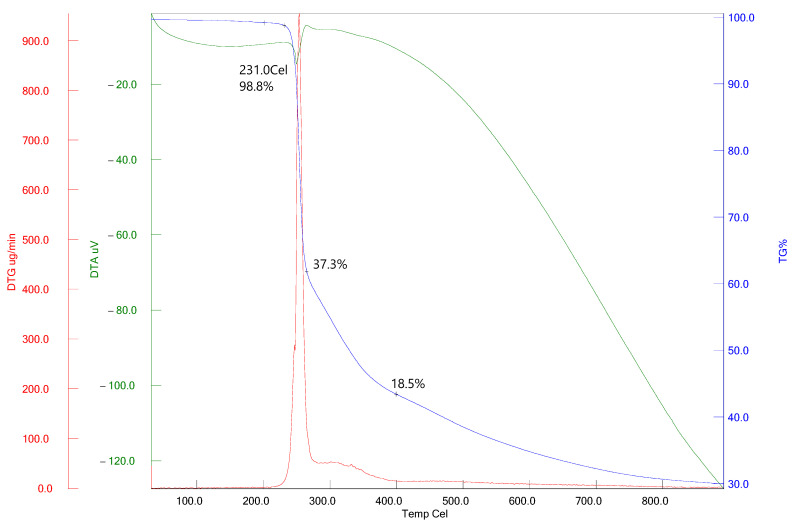
Studying the thermal properties of compound **2**.

Figure 6 displays the TGA, DTG, and DTA curves for compound **2**. The thermogravimetric (TGA) curve indicates that the decomposition begins around 218 °C and follows a two-step process. The initial stage of weight reduction accounts for 37.3%, whereas the subsequent stage accounts for 18.5%. The weight loss experienced from a temperature of 25 °C to 900 °C amounts to 69.9%. The DTG curve of compound **2** exhibited two peaks that corresponded to the TGA curve. The initial stage is associated with the determination of the melting point, as evidenced by the presence of an endothermic peak at 231 °C recorded on the DTA curve. The second stage is linked to thermal deterioration, as evidenced by the exothermic peak at 267 °C observed on the DTA curve [64].

### 3.6. Investigating the Kinetics of Isatin-Thiosemicarbazones

**Figure 7:**
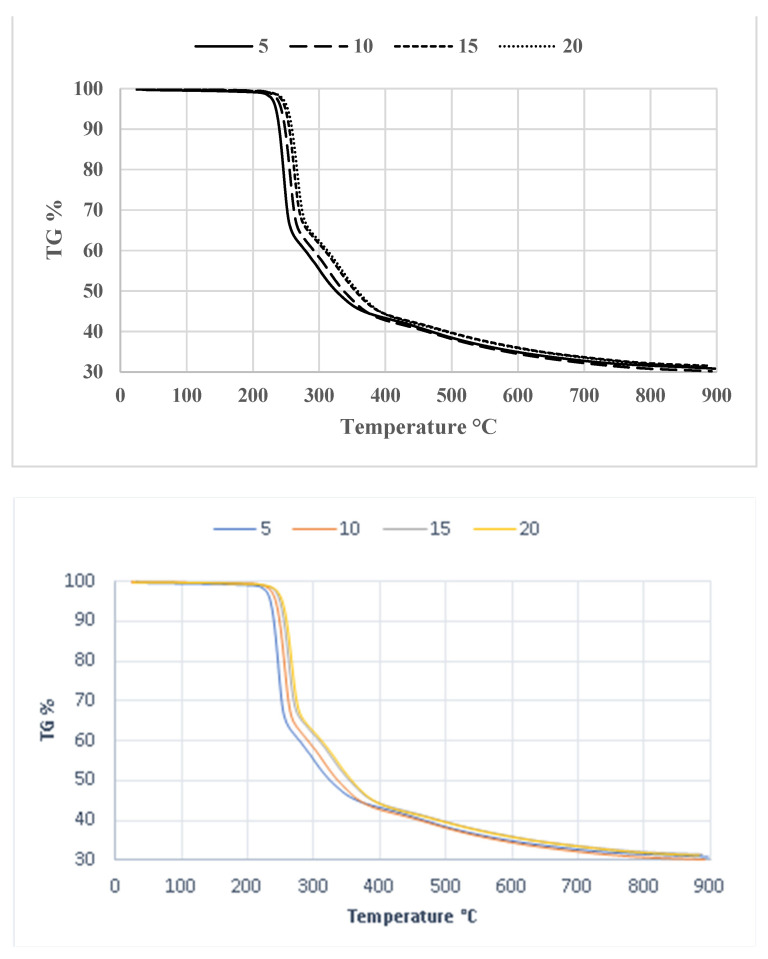
TGA curves of compound **1** at different heating rates.

The TGA curves of compound **1** are displayed in Figure 7, depicting four continuous heating rates: 5, 10, 15, and 20 °C min^−1^. As the heating rate increases from 5 to 20 °C, the TGA curves exhibit a rightward shift. Additionally, the peak temperature also changes towards a higher value with an increasing heating rate. Substantial reductions in weight are seen across all measures within the temperature range of 200–400 °C. Thermal decomposition occurs within the temperature range of 200 to 400 °C. Given that this phase has also been detected in the sample and its decomposition happens within the temperature range under investigation, it unavoidably impacts the kinetic curves [60].

**Figure 8:**
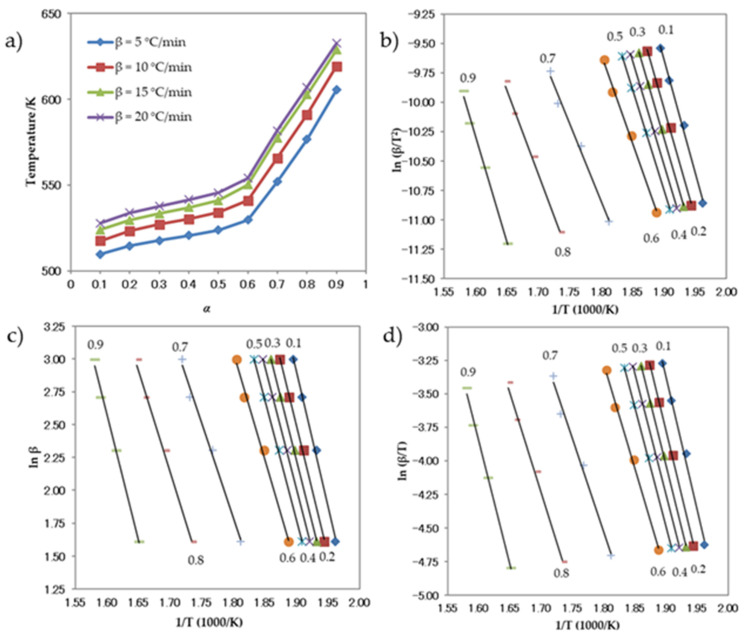
(**a**) Relationship between α and thermal decomposition temperature of the compound **1** at different heating rates, and Arrhenius plots for the (**b**) Kissinger, (**c**) Ozawa, and (**d**) Boswell methods.

Figure 8a depicts the temperature–response rate relationship for compound **1**, as represented by the curves. Figure 8b–d display the diagrams used to calculate the activation energy using the Kissinger, Ozawa, and Boswell approaches, respectively. The activation energies obtained from the three approaches exhibit a high degree of similarity. The standard deviations for each individual approach, as well as for the Kissinger, Ozawa, and Boswell methods, are presented in Table 7. The activation energy values for the thermal decomposition process were determined using three different methods: the Kissinger approach yielded a value of 137.60 kJ mol^−1^, the Ozawa method yielded a value of 146.58 kJ mol^−1^, and the Boswell technique yielded a value of 142.09 kJ mol^−1^. These calculations were performed under non-isothermal circumstances. Based on these results, it can be concluded that the activation energy needed for the decomposition of compound **1** falls within the range of 137–147 kJ mol^−1^.

**Figure 9:**
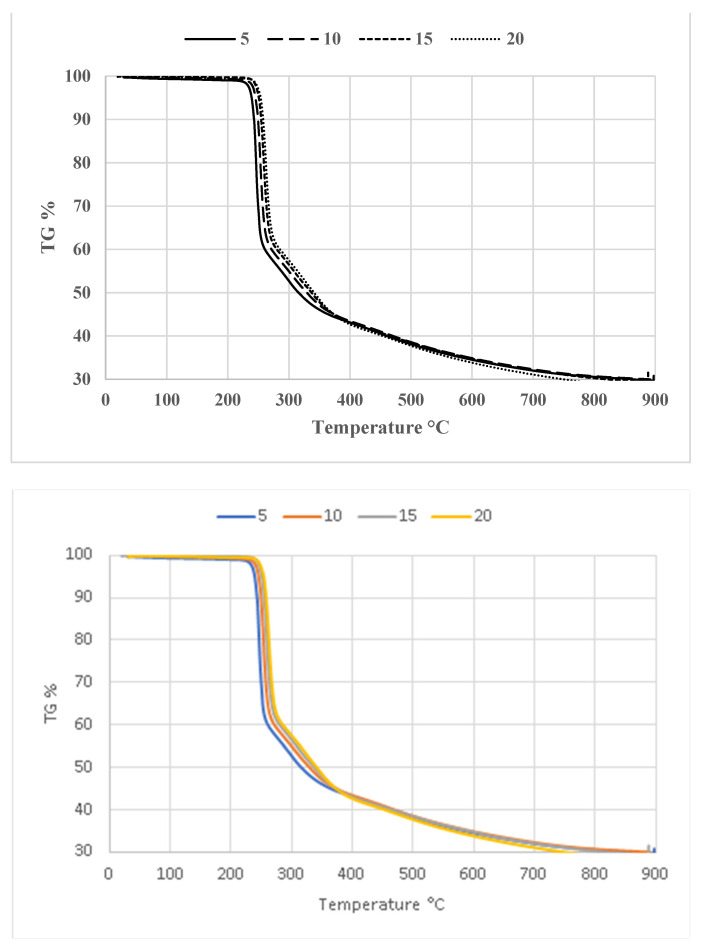
TGA curves of compound **2** at different heating rates.

**Figure 10:**
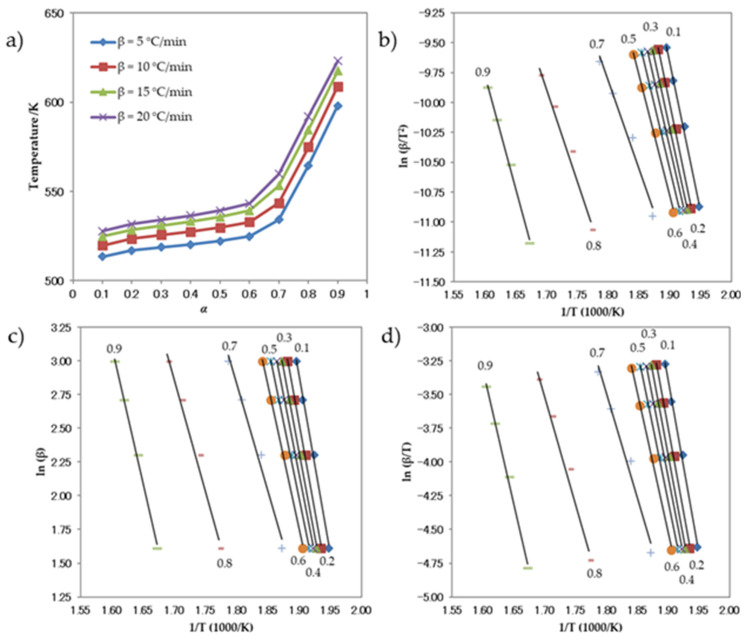
(**a**) Relationship between α and thermal decomposition temperature of compound **2** at various heating rates, and Arrhenius plots for the (**b**) Kissinger, (**c**) Ozawa, and (**d**) Boswell methods.

Figure 9 displays the thermogravimetric (TGA) curves of compound **2** obtained under several heating rates. Figure 9 demonstrates that the TGA curves shifted towards higher temperatures as the heating rates increased. The temperature–α curves for compound **2** are shown in Figure 10a. The graphs illustrating the calculation of activation energy using the Kissinger, Ozawa, and Boswell approaches are displayed in Figure 10b–d, respectively. The activation energy was determined using the same methodologies for all three procedures. Table 8 displays the results and standard deviations for each specific methodology, as well as for the Kissinger, Ozawa, and Boswell methodologies. The activation energies for the thermal decomposition of compound **2**, as determined by the Kissinger, Ozawa, and Boswell techniques, were 173.61, 182.48, and 178.04 kJ mol^−1^, respectively. Based on these figures, the amount of energy needed to decomposition compound **2** is between 173 and 183 kJ mol^−1^.

The non-isothermal kinetic investigations of isatin-thiosemicarbazone derivatives revealed that the activation energy needed for the thermal decomposition of compound **1** is lower than that needed for the thermal decomposition of compound **2**. The thermal stability of compounds is directly proportional to their activation energy; a higher activation energy value indicates more excellent thermal stability [57]. Based on the findings of non-isothermal decomposition kinetics, compound **2** exhibits more incredible activation energy than compound **1**. Compound **2** has superior heat stability. Additionally, it is recognized that the molecule with a lower activation energy exhibits superior antioxidant capabilities [58]. Therefore, it has been verified that compound **1**, which shows a greater yield, possesses superior antioxidant characteristics.

## 4. Conclusions

Biofuel is cheaper and greener than diesel. Biodiesel’s instability was studied because of its significant oxidation risk. Our study results are summarized below. 

The DSC observed natural and synthetic antioxidants’ heat crystallization. Many samples crystallized at │−11.76│–│−13.97│ °C. Analysis at lower temperatures purifies the material. The crystallization origin was dictated by temperature. Our research examined samples and temperatures. The measured temperature for B30D70 was somewhat lower than that for D100 at −11.76 °C, at −11.87 °C. The temperature for B30D70TROLOX was −12.03 °C, while that for B30D70–**2** was −12.73 °C. The coldest sample was B30D70–**1** (−13.97 °C). The results show the study samples’ temperatures. B30D70TROLOX, B30D70–**2**, and B30D70–**1** crystallize below D100. Antioxidants slow oxidation. Fast oxidation, early crystallization, and high oxidation susceptibility define the material. Sequential feature stability rises. Schiff bases **1** and **2** crystallized gasoline blends at a rate of 87.78% and 72.16%, respectively. D100, B30D70, B30D70TROLOX, B30D70–**2**, and B30D70–**1** oxidized at 25–275 °C, according to TGA. Deterioration occurred first. Thermal dynamics causes mass loss at certain temperatures. Recent investigations show rates of mass loss of 98.09% to 99.44% in samples. Biodiesel–diesel samples had small Tonset discrepancies that fit the curves. 

Sample functional groups were identified using FT-IR. B30D70–**2** and B30D70–**1** contained fewer functional groups than B30D70TROLOX. This reduction was caused by the 3000 ppm content of Schiff bases. Schiff bases increase composite oxidative stability, researchers have discovered. 

A DPPH˙ free radical activity assay tested the compounds’ antioxidant capabilities. The studies included spectrophotometry and scavenging. Isatin-thiosemicarbazones have free radical scavenging activity, with IC_50_ values of 66.178 ± 0.11 μM for DPPH˙ and 79.927 ± 0.13 μM. Compound **1** has the best antioxidant activity among the compounds, but this is less than that of Trolox. The chemical structure and behavior were explored. This study examined the substituents. Isatin-thiosemicarbazones enhanced biodiesel, a higher-efficiency diesel, in an antioxidant manner. 

The activation energies of the isatin-thiosemicarbazone derivative’s thermal breakdown were also assessed. We examined non-isothermal thermogravimetric (TGA) curves at 5, 10, 15, and 20 °K min^−1^. The Kissinger, Ozawa, and Boswell methods produced compounds **1** and **2** at activation energies of 137–147 and 173–183 kJ mol^−1^. Low-activation energy molecules had better antioxidant activity.

## Figures and Tables

**Table 1:** Physical data of the synthesized compounds (**1**–**2**).

Compound Code	R	Molecular Formula	Molecular Weight	Melting Point (°C)	Yield %
**1**	2-OCH_3_	C_17_H_16_N_4_O_3_S	356.41	218–220	87
**2**	2-F	C_16_H_13_FN_4_O_2_S	344.37	234–235	82

**Table 2:** Exploring the contents and codes of blends containing biodiesel, diesel, antioxidants, and isatin-thiosemicarbazones.

Sample	Biodiesel (%)	Diesel (%)
D100	-	100
B20D80	20	80
B20D80TROLOX	20	80
B20D80–**2**	20	80
B20D80–**1**	20	80

**Table 3:** Crystallization onset temperatures (°C) of D100, B20D80, B20D80TROLOX, B20D80–**2**, and B20D80–**1** determined using DSC in a N_2_ atmosphere *.

Sample	Crystallizations Onset Temperature (xi) (°C)	${\left(xi-x_{mean}\right)}^{2}$
D100	7.97	4.55
B20D80	8.48	2.64
B20D80TROLOX	10.89	0.62
B20D80–**2**	11.53	2.03
B20D80–**1**	11.65	2.39

* Values are expressed as means (*n* = 2).

**Table 4:** Thermogravimetric analysis (TGA) of samples.

Sample Name	Temperature Range (°C)	Max, Degradation Temperature (yi) (°C) (Tonset)	${\left(yi-y_{mean}\right)}^{2}$	Mass Loss (zi) (%)	${\left(zi-z_{mean}\right)}^{2}$
D100	25–250	99.36	0.06	11.52	5.29
B20D80	25–250	99.12	1.10^−4^	7.72	2.25
B20D80TROLOX	25–250	99.27	0.03	7.71	2.28
B20D80–**2**	25–250	99.24	0.02	8.94	0.08
B20D80–**1**	25–250	98.56	0.30	10.20	0.86

**Table 5:** Experimental FT-IR values of the compounds (cm^−^^1^).

Compound	ν(O–H)	ν(C–H) Aromatic	ν(C–H) Aliphatic	ν(C=O)	ν(N–H)	ν(C–N)	ν(C–O)
B20D80–**2**	–	2925	2874	1746	1458	1199	1079
B20D80–**1**	–	2925	2874	1746	1458	1173	1079
B20D80 TROLOX	3687	2925	2857	1746	–	–	1079
B20D80	3687	2925	2857	1746	–	–	1079
D100	3687	2925	2857	1746	–	–	1079

**Table 6:** Free radical scavenger effect of the compounds (μM) *.

Compound	IC_50_ Values μM
**1**	66.178 ± 0.11 ^b^
**2**	79.927 ± 0.13 ^c^
**Trolox**	8.757 ± 0.07 ^a^

*, ^a–c^ Values are expressed as means (*n* = 3).

**Table 7:** Calculating the relationship between activation energy values and α using three methods for compound **1** *.

α	Activation Energies (kJ mol^−1^)			The Standard Deviation(s) for the Kissinger, Ozawa, and Boswell Methods
	Kissinger	Ozawa	Boswell	
0.1	163.05	171.68	167.37	4.31
0.2	155.18	163.89	159.53	4.35
0.3	152.00	160.77	156.38	4.38
0.4	146.72	155.54	151.13	4.41
0.5	143.70	152.59	148.15	4.44
0.6	130.64	139.65	135.14	4.50
0.7	114.96	124.38	119.67	4.71
0.8	123.40	133.24	128.32	4.92
0.9	153.22	163.51	158.36	5.14
Standard deviation for each method	16.09	15.88	15.98	

* Values are expressed as means (*n* = 2).

**Table 8:** Calculating the relationship between activation energy values and α using three methods for compound **2** *.

α	Activation Energies (kJ mol^−1^)			The Standard Deviation(s) for the Kissinger, Ozawa, and Boswell Methods
	Kissinger	Ozawa	Boswell	
0.1	211.74	220.40	216.07	4.33
0.2	206.48	215.19	210.83	4.35
0.3	199.63	208.38	204.01	4.37
0.4	190.60	199.38	194.99	4.39
0.5	182.53	191.35	186.94	4.41
0.6	169.41	178.29	173.85	4.44
0.7	125.89	134.98	130.44	4.54
0.8	129.56	139.17	134.36	4.80
0.9	160.42	170.57	165.49	5.07
Standard deviation for each method	31.56	31.24	31.39	

* Values are expressed as means (*n* = 2).

## Data Availability

Data are contained within this article.
